# Effect of small extracellular vesicles derived from IL-10-overexpressing mesenchymal stem cells on experimental autoimmune uveitis

**DOI:** 10.1186/s13287-022-02780-9

**Published:** 2022-03-07

**Authors:** Yongtao Li, Xinjun Ren, Zhihui Zhang, Yanan Duan, Huan Li, Shuang Chen, Hui Shao, Xiaorong Li, Xiaomin Zhang

**Affiliations:** 1 Tianjin Key Laboratory of Retinal Functions and Diseases, Tianjin Branch of National Clinical Research Center for Ocular Disease, Eye Institute and School of Optometry, Tianjin Medical University Eye Hospital, Tianjin, China; 2 Department of Ophthalmology and Visual Sciences, Kentucky Lions Eye Center, University of Louisville, School of Medicine, Louisville, KY USA

**Keywords:** Autoimmune uveitis, Interleukin 10, Mesenchymal stem cell, Small extracellular vesicle, T-cell

## Abstract

**Background:**

Autoimmune uveitis is a sight-threatening intraocular inflammation mainly caused by immune dysregulation. The development of safe and effective therapeutic approaches is urgently needed. Small extracellular vesicles (sEVs) derived from mesenchymal stem cells (MSCs) have been demonstrated to inhibit autoimmune responses; however, the immunosuppressive effect of MSC-sEVs is too weak for clinical transfer. In the current study, we investigated the therapeutic effect of IL-10-overexpressing MSC-sEVs (sEV-IL10) on experimental autoimmune uveitis (EAU) and studied the underlying mechanism.

**Methods:**

Mice were randomly grouped and received a single tail vein injection of different sEVs (50 μg) or PBS on day 11 post-immunization. The clinical and histological scores were graded, and the percentage of T helper cell was measured. To investigate the effect of sEVs on the proliferation of T-cells and the differentiation of Th1, Th17 and Treg cells, T-cells were cocultured with sEVs under the corresponding culture conditions. After labeled with PKH-26, sEVs were traced both in vivo and in vitro.

**Results:**

Compared with normal or vector sEV-treated groups, mice in the sEV-IL10-treated group had lower clinical and histological scores with lower percentages of Th1 and Th17 cells in the eyes and higher percentages of Treg cells in the spleen and draining lymph nodes (LN). Furthermore, sEV-IL10 enhanced the suppressive effect of MSC-sEVs on the proliferation of T-cells and differentiation of Th1 and Th17 cells, whereas upregulated the differentiation of Treg cells. Both in vivo and in vitro experiments demonstrated that MSC-sEVs were rapidly enriched in target tissues and internalized by T-cells.

**Conclusion:**

These results suggested that sEV-IL10 effectively ameliorates EAU by regulating the proliferation and differentiation of T-cells, indicating sEVs as a potential novel therapy for autoimmune uveitis or other autoimmune diseases.

**Supplementary Information:**

The online version contains supplementary material available at 10.1186/s13287-022-02780-9.

## Background

Autoimmune uveitis is one of the leading causes of blindness owing to its frequent recurrence. Corticosteroids and immunosuppressive agents are the most commonly prescribed drugs for the treatment of uveitis [1]. However, their clinical applications have been limited by long-term systemic side effects and the unresponsiveness of some patients. Biological agents, especially anti-tumor necrosis factor-α (TNF-α) monoclonal antibodies, have been reported to be effective in some refractory cases; however, they are limited to patients at high risk of infection [2, 3]. Therefore, there is an urgent need to develop more effective and safer improved therapeutic approaches.

Mesenchymal stem cells (MSCs) are adult stem cells with the capacity for multilineage differentiation and self-renewal. In particular, MSCs have strong immunosuppressive functions and can suppress various immune responses, including autoimmunity and allograft rejection, without weakening anti-infection immunity [4, 5]. Previous studies have demonstrated that MSCs can significantly alleviate experimental autoimmune uveitis (EAU) in animal models [4, 6–11]. However, cell-based therapy not only raises the challenge of quality control regarding the preservation and transport of cell products, but also increases the risk of vessel obstruction, malignant transformation, and allogenic immunological rejection [12–15].

Exosomes are tiny vesicles (ranging from 40 to 150 nm in diameter) with a lipid bilayer membrane structure containing functional molecules, such as proteins, mRNAs, microRNAs, and DNAs. Considering that current separation protocols cannot completely remove nonexosome vesicles and lack evidence of the endosomal biogenesis pathway, the term small extracellular vesicles (sEVs) has been recommended [16]. Accumulating evidence have suggested that sEVs mediate the paracrine pathway of MSCs, exerting biological functions similar to parent cells [17–19]. Compared with cells, sEVs do not have the ability to replicate, differentiate, or mutate, and are easy to store and transport [20]. In addition, as sEVs been show to mediate intercellular communication though transferring their cargo to recipient cells, and their lipid bilayer membrane can protect the enwrapped proteins and miRNAs from degradation, sEVs have been widely used as drug carriers [21, 22]. Therefore, as a novel type of cell-free therapy, sEV-based therapy is expected to be a desirable surrogate for stem cell therapy [23]. Our previous studies revealed that MSC-derived sEVs (MSC-sEVs) could inhibit EAU; however, this involved the administration of large doses and frequent periocular injections [24].

Interleukin 10 (IL-10) is a major immunoregulatory cytokine that contributes to immune homeostasis and is a key factor mediating the immunosuppression of MSCs [25]. However, the application of IL-10 for the treatment of autoimmune diseases has not been successfully translated into clinical practice because of its short half-life in vivo [26]. Therefore, in this study, we utilized MSC-sEVs as carriers for IL-10 to protect it from degradation and enhance its half-life in vivo [26]. We demonstrated that IL-10-overexpressing MSC-sEVs (sEV-IL10) dramatically enhanced the therapeutic effect of MSC-sEVs on EAU by suppressing the proliferation of uveitogenic T-cells and differentiation of Th1/Th17 cells and promoting the differentiation of Treg cells. We also found that sEV-IL10 were internalized by T-cells in vitro and rapidly enriched in target tissues in EAU mice after intravenous injection (i.v.).

## Methods

### Animals

All animal procedures conformed to the statement for the use of animals in ophthalmic and vision research of the Association for Research in Vision and Ophthalmology (ARVO) and approved by the Laboratory Animal Care and Use Committee of Tianjin Medical University Eye Hospital (TMUEC) (No. TJYY2019103022). Female C57BL/6 J mice (B6) (7-week-old) were purchased from Vital River Experimental Animal Center (Beijing, China) and housed under specific pathogen-free (SPF) conditions in the animal center of TMUEC at 21–25 °C and 45–65% humidity with a 12 h day/dark cycle.

### Isolation and culture of mesenchymal stem cells

Human umbilical cord MSCs were provided by Beilai Biological Co., Ltd. (Beijing, China). The isolation and culture of MSCs were performed as previously reported [24]. MSCs were identified by their capacity to differentiate into adipocytes, chondrocytes, and osteocytes when cultured under appropriate conditions in vitro [24]. Further characterization was based on the expression of the CD90, and CD29 markers, and the absence of the CD34 and CD45 markers, as previously described [24].

### Production and transduction of lentiviruses

Briefly, IL-10-overexpressing or empty vector plasmids (pCDH-CMV-MCS-EF1-copGFP) (Hanbio Biotechnology, Shanghai, China) were transfected into human embryonic kidney 293 T cells (HEK-293 T) along with PMD2G and PSPAX2 (Hanbio Biotechnology) using lipofectamine 2000 (Invitrogen, California, USA) according to the manufacturer’s instructions. Lentiviruses in the culture supernatant were collected at 48 and 72 h after transfection and filtered (0.22 μm). The supernatant was concentrated by ultracentrifugation at 72 000 × *g* for 2 h, and the viral titer was then determined using the serial dilution method [27].

### Transfection and supernatant collection

MSCs at passage 2 grown to 60% confluency were infected with lentivirus with a multiplicity of infection (MOI) of 50 and incubated with 8 μg/mL polybrene (Sigma-Aldrich, St. Louis, USA) for 8 h. MSCs from passages 3–5 were used for the production of sEVs. MSCs were cultured in complete DMEM/F-12 media (Gibco, California, USA) containing 10% FBS (Gibco) and 100 U/mL penicillin and streptomycin (Gibco). When cells fusion reached 60%, media were replaced with serum-free MSC XF Basal medium (BI, Kibbutz, Israel) supplemented with 0.6% MSC XF Supplement Mix (BI). After 48 h of incubation, the culture supernatants were harvested.

### Quantitative Real-time RT-PCR (qRT-PCR)

Total RNA was extracted using the TRIzol reagent (Invitrogen) according to the manufacturer’s instructions and transcribed into cDNA using a RevertAid First Strand cDNA Synthesis Kit (Thermo Fisher, Massachusetts, USA). Each qPCR reaction in 384-well plates contained FastStart SYBR Green Master (Roche, Basel, Switzerland), cDNA, and 0.25 μM forward and reverse primers. The relative levels of mRNA expression of target genes were calculated using the 2^−ΔΔCq^ method, with GAPDH as an internal standard [28]. The sequences of each primer were as follows: IL-10 forward, 5′-TGAAAACAAGAGCAAGGCCG-3′, reverse: 5′-ATAGAGTCGCCACCCTGATG-3′; GAPDH forward: 5′-AATGGGCAGCCGTTAGGAAA-3′, reverse: 5′ GCGCCCAATACGACCAAATC-3′.

### Isolation of small extracellular vesicles

The generated sEVs were purified using the ultracentrifugation method. CM was centrifuged at 200×*g* for 10 min at 4 °C, followed by centrifugation at 2000×*g* for 20 min at 4 °C to remove cell debris, and an additional round of centrifugation at 10 000×*g* for 30 min at 4 °C to remove apoptotic bodies. sEVs were harvested by ultracentrifugation twice at 110 000×*g* for 70 min at 4 °C. Pellets were further purified by resuspension in PBS (Gibco) and ultracentrifugation at 110 000×*g* for another 70 min at 4 °C to remove any contaminating proteins. All ultracentrifugation steps were performed on an Optima XLA/I centrifuge with an An-45Ti rotor (Beckman-Coulter, California, USA).

### Characterization of small extracellular vesicles

sEVs were characterized according to previously published protocols [24]. In brief, after fixation with 4% paraformaldehyde (Sigma-Aldrich), sEV sample was dropped on a carbon copper grid, stained with a 2% uranyl acetate solution (Sigma-Aldrich), and photographed using a Phillips CM10 electron microscope (Phillips Electron Optics, Eindhoven, The Netherlands). The size distribution of sEV samples was determined using a nanoparticle analysis system (NanoSight Ltd, Amesbury, UK). The expression of TSG101 and CD9 sEV surface markers was identified by western blotting. sEV samples (10 μg) were separated by polyacrylamide gel electrophoresis (SDS-PAGE) after heat denaturation at 95 °C for 5 min. Proteins were transferred onto PVDF membranes (Sigma-Aldrich) using a wet transfer electrophoresis tank at 90 V for 2 h and incubated in 5% skim milk in PBS supplemented with 0.05% Tween-20 for 1 h at 25 °C. Quantification of sEVs was performed using the BCA method.

### Induction and treatment of experimental autoimmune uveitis

For EAU induction, mice were subcutaneously immunized with an emulsion containing equal volumes of incomplete Freund’s adjuvant (IFA) (Sigma-Aldrich) with 5 mg/mL desiccated *Mycobacterium tuberculosis* (Sigma-Aldrich) and 250 μg IRBP_651–670_ (Hanhong group, Shanghai, China) in PBS, distributed at 4 spots on the tail base and flank. Mice were intraperitoneally administered 500 ng pertussis toxin (PTX) (List Biological Laboratories, California, USA) on the day of immunization and 24 h post-immunization.

First, mice were randomly grouped (*n* = 6 mice) and received i.v. injection of recombinant IL-10 or equal volume of PBS, respectively. Mice in single injection group received a single i.v. injection of 500 ng recombinant IL-10 in 200 μl PBS on day 11 post-immunization. Mice in multiple injection group received i.v. injection of 500 ng recombinant IL-10 in 200 μl PBS for 5 consecutive days (once a day from day 11 to day 15 post-immunization).

Next, mice were randomly grouped (*n* = 6 mice) and received a single tail vein injection of sEVs or PBS on day 11 post-immunization. For contrast experiments, mice were injected with 50 μg sEV-N, IL10-overexpressing MSC-sEVs (sEV-IL10), vector-infected MSC-sEVs (sEV-V), or equal volume of PBS, respectively.

### Clinical and histological assessment of experimental autoimmune uveitis

Clinical and histological grading was scored as described by Caspi et al. [29]. The retinal status was examined once every other day using an indirect binocular ophthalmoscope through a 90 D fundus lens starting on day 8 post-immunization. Mice were killed on day 18 post-immunization, and their eyeballs were collected and fixed in 4% paraformaldehyde. Fixed retinal tissues were embedded in paraffin and cut into 5-μm-thick sections. The sections were then stained with hematoxylin and eosin (H&E) according to standard procedures.

### Labeling and tracing of small extracellular vesicles

Purified sEVs were labeled using a PKH26 kit (Sigma-Aldrich) according to the manufacturer’s protocol. Labeled sEVs were washed twice in PBS by centrifuged at 110 000×*g* for 70 min.

To trace sEVs in vivo, 100 μg PKH-26 labeled sEVs was injected into EAU (11 d post-immunization) or naïve mice via the tail vein. Mice were killed at 24, 48, or 72 h post-injection. Tissue samples (eyeball, spleen, lymph node, heart, liver, and kidney) were collected immediately after killing and embedded in optimal cutting temperature (OCT) (Thermo Fisher) compound to obtain frozen sections (8 μm). Frozen sections were then fixed with paraformaldehyde, incubated with 5 mg/mL DAPI for nuclei staining, and visualized under a LSM510 confocal microscope (Zeiss, Oberkochen, Germany).

To trace sEVs in vitro*,* purified CD4 + T-cells were isolated from spleens of B6 mice using a negative CD4 + T-cell isolation kit (Invitrogen) and cultured in complete RPMI (Gibco) in a 96-well plate precoated with 10 μg/mL anti-CD3 plus 1 μg/mL anti-CD28 (BioLegend, London, UK). After 24 h of incubation, 20 μg/mL PKH-26 labeled sEVs were added. CD4 + T-cells were collected after 1, 3, and 6 h of coculture with sEVs and then spread on an adhesive glass slide. Cells were fixed with 4% paraformaldehyde for 30 min and blocked with PBS containing 5% normal goat serum and 0.3% Triton (Sigma) for 1 h. FITC-conjugated anti-CD4 Ab and DAPI (Sigma) were used to stain cellular membranes and nuclei, respectively. Immunofluorescence intensity was visualized using confocal microscopy (LSM510; Zeiss).

### Isolation and analysis of infiltrating cells

Eyeballs were enucleated under anesthesia on day 18 post-immunization. The optic nerve, cornea, and lens were removed. The remaining tissues were squashed through a 70-µm cell strainer. After centrifugation, enzyme digestion was performed using 1 mg/mL collagenase D (Sigma-Aldrich) for 45 min. Cells were centrifuged, washed with PBS, and resuspended in CM. Spleen and draining lymph nodes (cervical and mandibular lymph node) were also isolated under anesthesia on day 18 post-immunization. After squashed through a 70-µm cell strainer and lysed with red blood cell lysis buffer (Sigma), spleen and draining lymph nodes were prepared into single cell suspensions.

Part of cells were then stimulated with 50 ng/mL PMA (Sigma), 1 μg/mL ionomycin (Sigma), and 1 μg/mL brefeldin A (Abcam, Cambridge, USA) for 5 h. Finally, stimulated cells were stained for further intracellular cytokine analysis.

### T-cell proliferation assay

Purified CD4 + T-cells isolated from lymph nodes and spleens of naive mice using a negative CD4 + T-cell isolation kit (Miltenyi Biotec, California, USA). The purified CD4 + T-cells were then labeled with 1 μM CFSE (Invitrogen) for 10 min, and seeded in 96-well plates precoated with 10 μg/mL anti-mouse CD3 mAb and 2 μg/mL anti-mouse CD28 mAb (BioLegend) at a density of 5 × 10^5^ cells per well. Cell growths were analyzed by FACS at 72 h post-culture.

### Ex vivo T-cell recall assay

T-cells (4 × 10^5^ cells/well) were isolated from the draining lymph nodes (cervical and mandibular lymph node) and spleens of EAU mice on day 14 post-immunization through a nylon wool column and cocultured with syngeneic irradiated (30 Gy) APCs (4 × 10^5^ cells/well) in 96-well plates with graded doses of IRBP_651–670_ (0, 1, 10, and 20 μg/mL), in a total 200 μL volume. After 72 h of culture, the BrdU assay was performed to assess the proliferation of T-cells using the BrdU Cell Proliferation Assay Kit (Abcam). The IFN-γ and IL-17A concentrations in the cell culture supernatants (with 20 μg/mL IRBP_651–670_) were measured using ELISA kits according to the manufacturer’s instructions (R&D Systems).

### In vitro T-cell differentiation assay

Naïve CD4 + CD45RA + T-cells were enriched using an immunomagnetic CD4 + T cell isolation kit (Miltenyi Biotec). Cells were seeded in anti-CD3/CD28 precoated 96-well plates at a density of 2 × 10^5^ cells/well and cultured under Th1, Th17, and Treg polarizing conditions. For Th1 polarization, cells were cultured in X-Vivo15 serum-free medium (Lonza, Basel, Switzerland) supplemented with IL-12 (10 ng/mL) and anti-IL4 (10 μg/mL). For Th17 polarization, cells were cultured in X-Vivo15 serum-free medium supplemented with IL-6 (30 ng/mL), anti-IL4 (10 μg/mL), anti-IFN-γ (10 μg/mL), IL-23 (6 ng/mL), and TGF-β1 (2.5 ng/mL). For Treg polarization, cells were cultured in X-Vivo15 serum-free medium supplemented with TGF-β1 (5 ng/mL) and IL-2 (20 ng/mL). After 5 d of culture, cells were collected and analyzed by FACS. Recombinant cytokines were purchased from R&D Systems (Minneapolis, USA), while antibodies against these cytokines were purchased from BD Biosciences (California, USA).

### Flow cytometry analysis

Cells were stained with fluorescein-conjugated anti-mouse CD4 and washed twice with wash buffer (PBS plus 2% BSA). For intracellular staining, fixation and permeabilization of cells were further preformed. Then, the samples were intracellular stained for fluorescein-conjugated antibodies specific for IFN-γ, IL-17A, and Foxp3 in permeabilization wash buffer. Unless otherwise noted, antibodies used for staining were purchased from BioLegend. Flow cytometric analysis was performed with a BD FACSCalibur flow cytometry, and acquired data were analyzed by FlowJo software (Oregon, USA).

### Statistical analysis

All experiments were independently repeated 3 times. All data were analyzed using the version 22.0 SPSS software (IBM, Chicago, USA) and presented as the mean ± SD. Unpaired Student’s *t*-test was used to assess the significance between 2 groups. Experimental data for multiple group comparisons were analyzed using one-way ANOVA. Differences were considered statistically significant at *P* < 0.05.

## Results

### Characterization of mesenchymal stem cell-small extracellular vesicles

Prior to using isolated MSCs, we characterized cells for the positive expression of the CD90, and CD29 markers, and negative expression of the CD34 and CD45 markers, as well as for their multilineage differentiation potential (Additional file 1: Fig. S1).

**Fig. 1:**
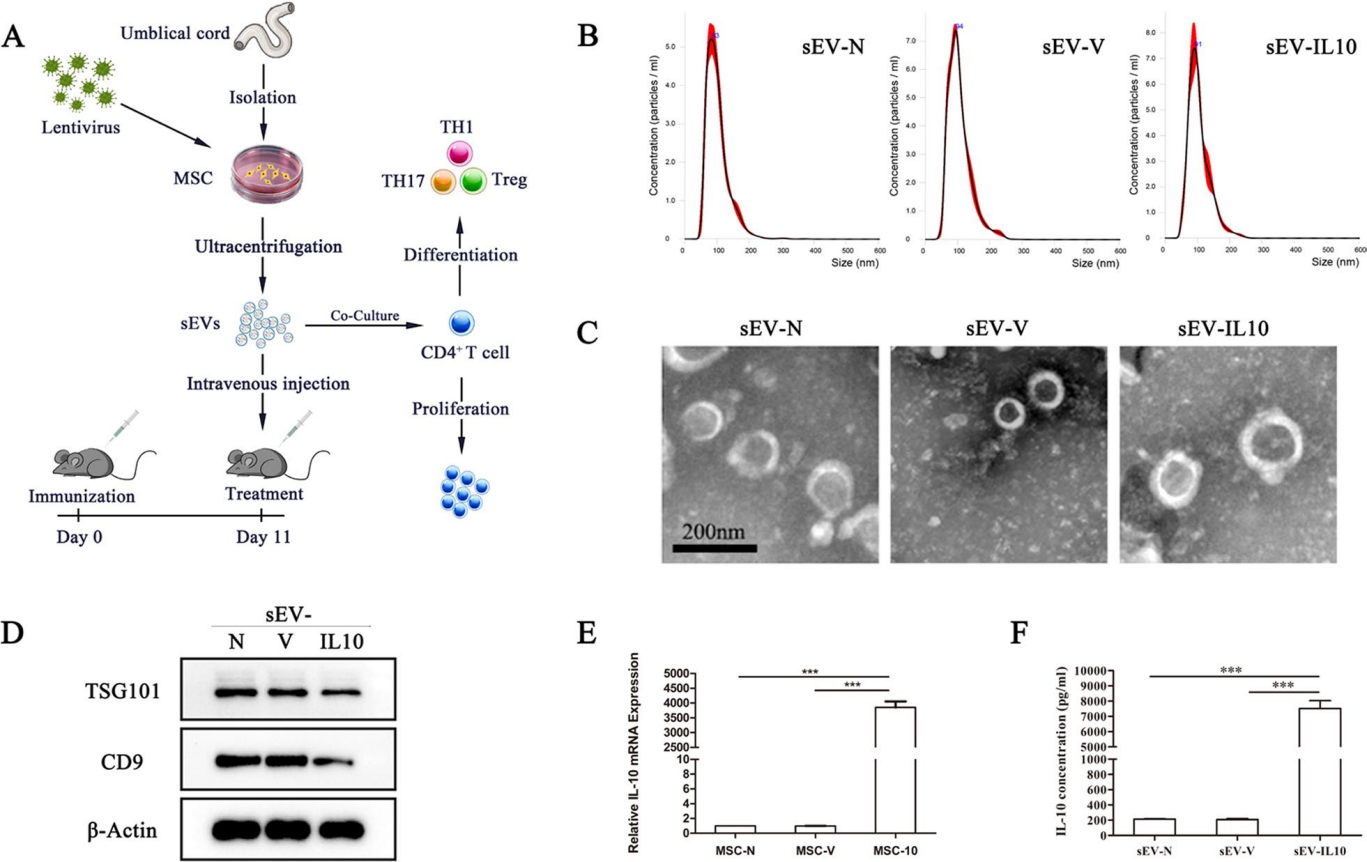
Characterization of MSC-derived sEVs. **A** Schematic illustration of the experimental workflow. **B** Analysis of sEV sizes from each group using Nanosight. **C** Transmission electron microscopy images of sEVs from each group. Scale bar = 200 nm. **D** Western blotting showing the expression of TSG101 and CD9 exosomal markers in sEVs. **E** Validation of the expression of the *IL-10* gene in lentiviral-infected (IL-10-overexpressing, vector) and normal (MSC-N) MSCs using quantitative PCR. **F** The expression of the IL-10 protein in sEVs from each group was detected using ELISA analysis. Mean ± SD, *n* = 3 in triplicates, one-way ANOVA test. ***P < 0.01

We also evaluated the diameter distribution of purified sEVs from MSCs using Nanosight; we found that the average size of sEVs in the sEV-N, sEVIL-10, and sEV-V groups was close to 100 nm, and the diameter of all sEVs exhibited a relatively narrow size distribution with nearly 90% of vesicles having a diameter of less than 150 nm (Fig. 1B). Transmission electron microscopy revealed that the sEVs in the sEV-N, sEV-IL10, and sEV-V groups showed a cup-shaped morphology with a lipid bilayer structure, with the diameters of most sEVs being approximately 80–120 nm (Fig. 1C). Western blotting confirmed that all sEV samples expressed the TSG101 and CD9 tetraspanin markers (Fig. 1D). Successful transfection of the MSCs with lentivirus was initially confirmed 48 h post-transfection by fluorescence microscopy (Additional file 2: Fig. S2). Real-time PCR revealed that the MSCs transfected with IL-10-expressing lentivirus expressed high levels of *Il-10* mRNA (Fig. 1E). In addition, sEVs derived from IL-10-overexpressing MSCs expressed high levels of the IL-10 protein, as indicated by ELISA (Fig. 1F).

### Intravenous injection of small extracellular vesicles derived from IL-10-overexpressing MSCs dramatically enhanced the therapeutic effect on experimental autoimmune uveitis

**Fig. 2:**
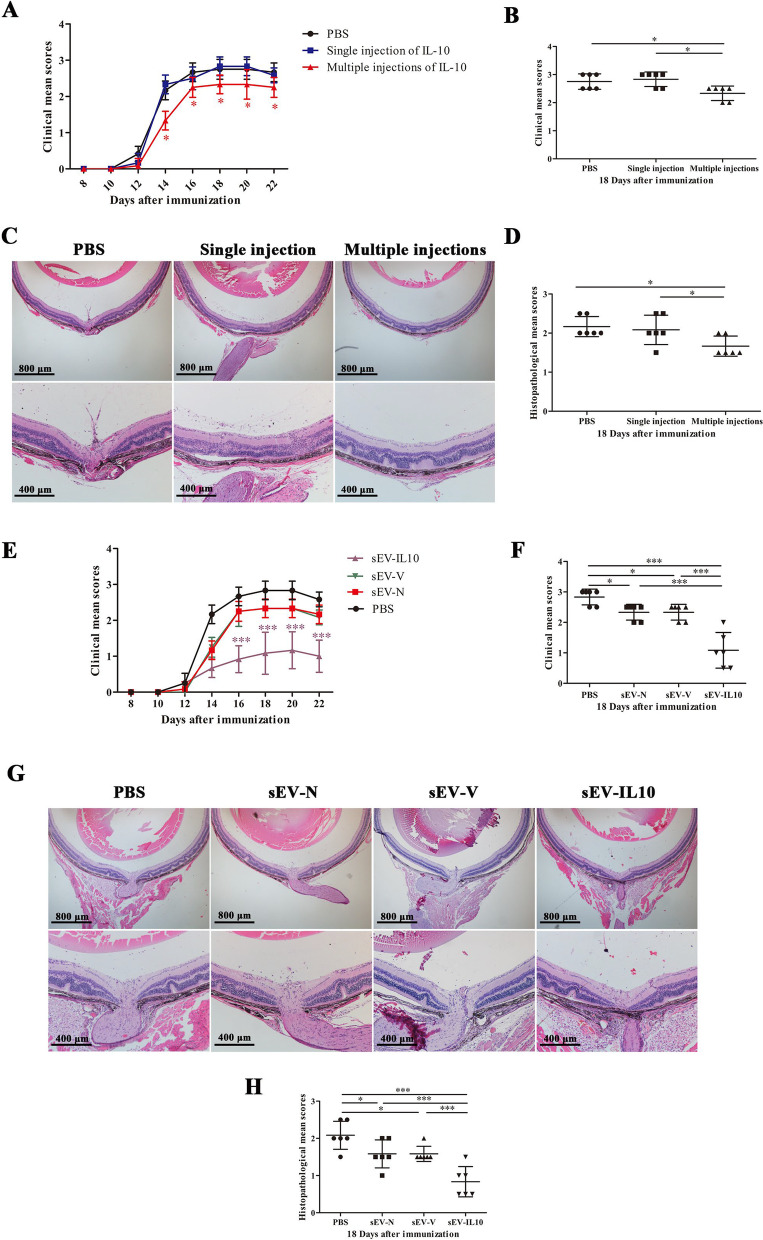
Treatment with sEV-IL10 dramatically enhanced the therapeutic effect of sEV-N. **A** Mean clinical scores of mice in single injection group and multiple injection group recorded every 2 d from day 8 to day 22 post-immunization. **B** Mean clinical scores of mice in single injection group and multiple injection group on day 18 post-immunization. **C**, **D** Representative histopathological images and scores in the single injection group and multiple injection group. **E** Mean clinical scores in the sEV-N-, sEV-IL10-, and sEV-V-treated (50 μg) groups recorded every 2 d from day 8 to day 22 post-immunization. **F** Mean clinical scores of each sEV-treated group on day 18 post-immunization. **G**, **H** Representative histopathological images and scores in the sEV-N-, sEV-IL10-, and sEV-V-treated (50 μg) groups. Mean ± SD, *n* = 6 per group, one-way ANOVA test. ***P < 0.01, *P < 0.05

We first examined the therapeutic effect of recombinant IL-10 in EAU mice. Mice received a single i.v. injection of 500 ng recombinant IL-10 on day 11 post-immunization or five days of consecutive i.v. injections of recombinant IL-10 starting on day 11 post-immunization. We found that only consecutive injection of recombinant IL-10 reduced the severity of EAU, with a significant reduction of clinical scores from day 14 to day 22 post-immunization (*P* < 0.05) (Fig. 2A, B). The histological scores at the peak of EAU (day 18 post-immunization) were in accordance with the clinical scores (Fig. 2C, D).

Next, we compared the therapeutic effects of sEVs in the sEV-N, sEV-IL10, and sEV-V groups on EAU. To this end, we i.v. injected 50 μg sEV-N, sEV-IL10, or sEV-V into EAU mice on day 11 post-immunization. We found that the mean clinical score of the sEV-IL10-treated group was significantly lower than other groups from day 14 to day 22 post-immunization (*P* < 0.01) (Fig. 2E, F). We did not observe any significant differences between the sEV-N and sEV-V groups. We further noticed that the histological scores at the peak of EAU (day 18 post-immunization) were in accordance with the clinical scores (Fig. 2G, H). We specifically observed that mice in the sEV-IL10-treated group showed fewer infiltrating inflammatory cells in the eye, less photoreceptor layer damage, and milder retinal folds than mice in the other groups.

**Fig. 3:**
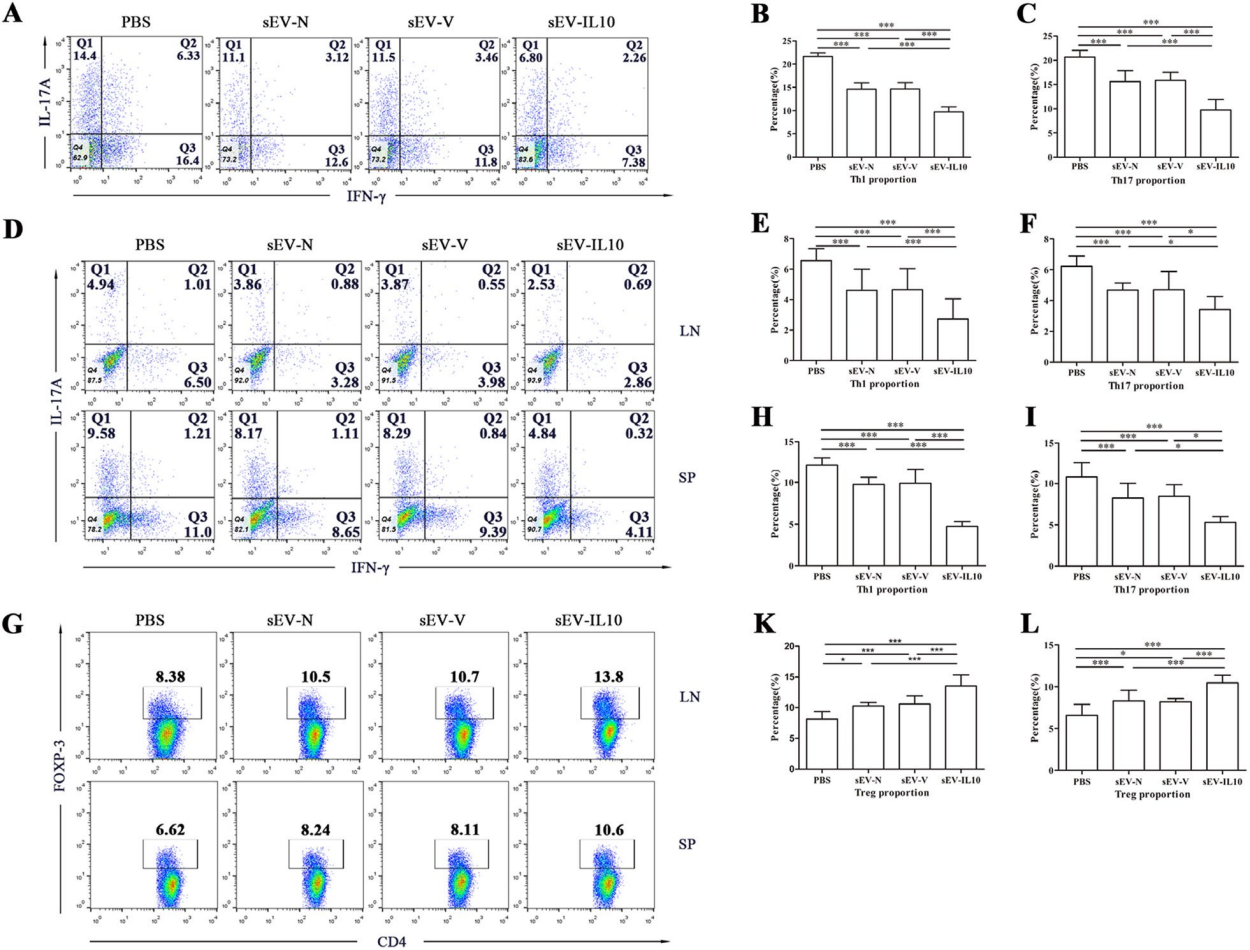
Treatment with sEV-IL10 results in a significant reduction in the infiltration of Th1 and Th17 cells in the eye, whereas in an increment of Treg cells in the spleen and draining lymph nodes. **A–C** Flow cytometric analysis of CD4 + IFN-γ + and CD4 + IL17A + cells in the eyeball from each group. **D–I** Flow cytometric analysis of Th1 (CD4 + IFN-γ +) and Th17 (CD4 + IL17A +) cells in the draining lymph nodes and spleen from each group. **G–L** Flow cytometric analysis of CD4 + FOXP3 + cells in the draining lymph nodes and spleen from each group. Mean ± SD, *n* = 6 per group, one-way ANOVA test. ***P < 0.01, *P < 0.05

Th1, Th17, and Treg cells play crucial roles in the pathogenesis of EAU. Hence, we examined the T-lymphocyte subsets in the eyes, spleens, and draining lymph nodes (LNs) of mice in each group at the peak of EAU using flow cytometry. We detected that mice in the sEV-N and sEV-V groups had lower percentages of CD4 + IFN-γ + and CD4 + IL17 + cells in the eyes, and higher percentages of CD4 + FOX3 + cells in the spleen and LNs than the PBS control group. In contrast, we noticed that administration of sEV-IL10 further reduced the infiltration of CD4 + IFN-γ + and CD4 + IL17 + cells in the eyes (Fig. 3A–C), spleen and LNs (Fig. 3D–I), whereas upregulated CD4 + FOX3 + cells in the spleen and LNs (Fig. 3G–L).

### Small extracellular vesicles were rapidly enriched in target tissues in vivo and internalized by T-cells in vitro

**Fig. 4:**
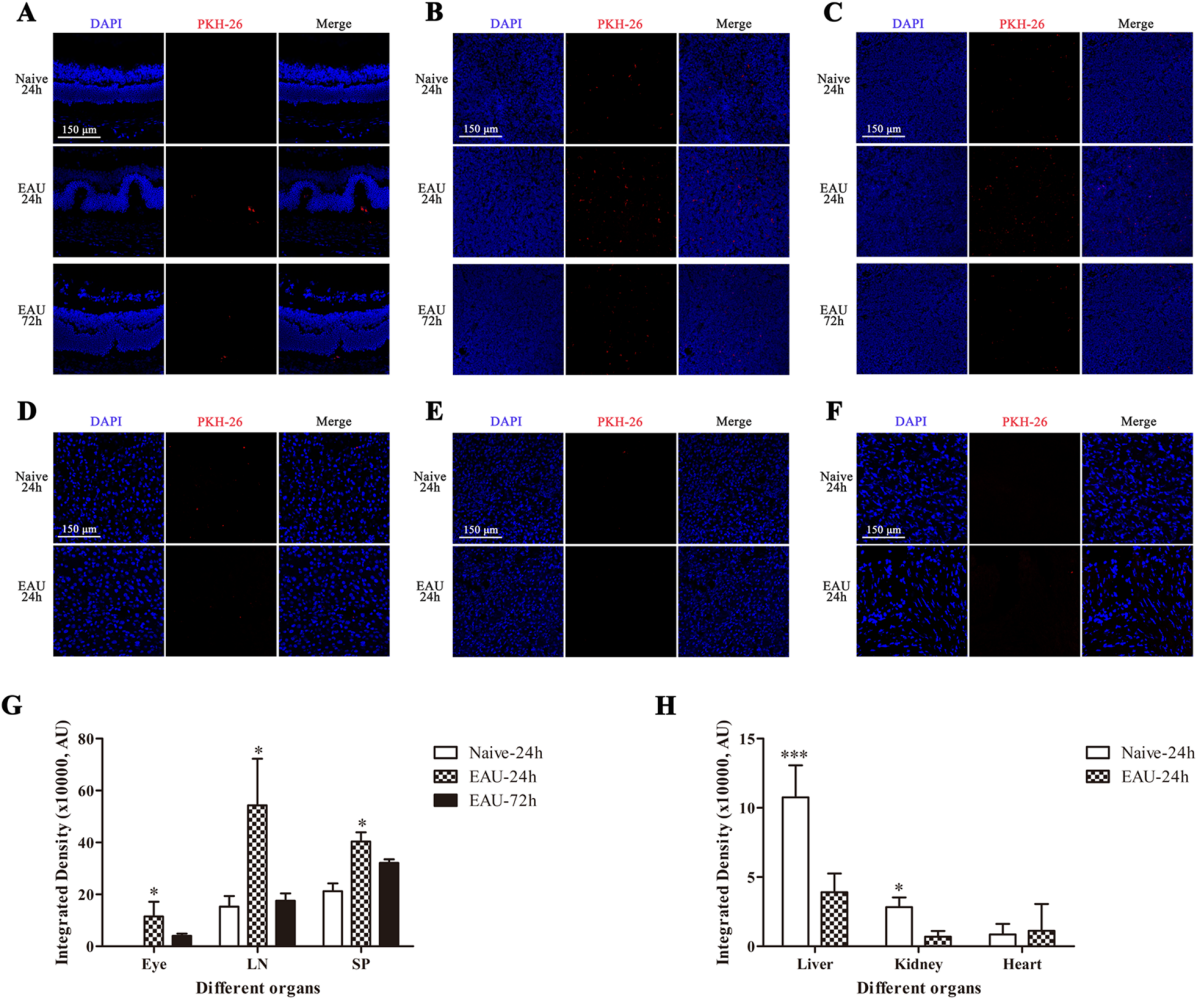
The in vivo distribution of intravenously injected sEVs in different organs in EAU and naive mice was examined using confocal microscopy. **A** Representative confocal images of PKH-26 immunofluorescence in the eyeballs of EAU and naive mice, which were i.v. injected with sEV-N (100 μg) 24 or 72 h prior. **B**, **C**. Representative confocal images of PKH-26 immunofluorescence in the lymph nodes and spleen of EAU and naive mice, which were i.v. injected with sEV-N (100 μg) 24 or 72 h prior. **D–F** Representative confocal images of PKH-26 immunofluorescence in the liver, kidney, and hearts of EAU and naive mice, which were i.v. injected with sEV-N (100 μg) 24 h prior. **G-H**. The fluorescence intensity was measured by integrated density using ImageJ software. Scale bar = 150 μm. Mean ± SD, *n* = 3 per group, one-way ANOVA test. ***P < 0.01, *P < 0.05

To observe the distribution of sEVs in target organs, we labeled sEVs with red fluorescence PKH-26, i.v. injected them into naïve and EAU mice, and traced them in vivo. After 24 and 72 h, we collected the eyeballs, spleens, LNs, livers, kidneys, and hearts of mice. We identified PKH-26-labeled red sEVs in the eyeballs of EAU mice at 24 h, which were still detected at 72 h, whereas no red fluorescence was observed in the eyes of naïve mice (Fig. 4A). We also detected the presence of red fluorescence in the spleen and LNs of EAU mice, which attenuated over time and was stronger than that in the spleen and LNs of naïve mice (Fig. 4B, C, G). Only a trace level of fluorescence was detected in the livers, kidney and heart of mice (Fig. 4D–F, H).

**Fig. 5:**
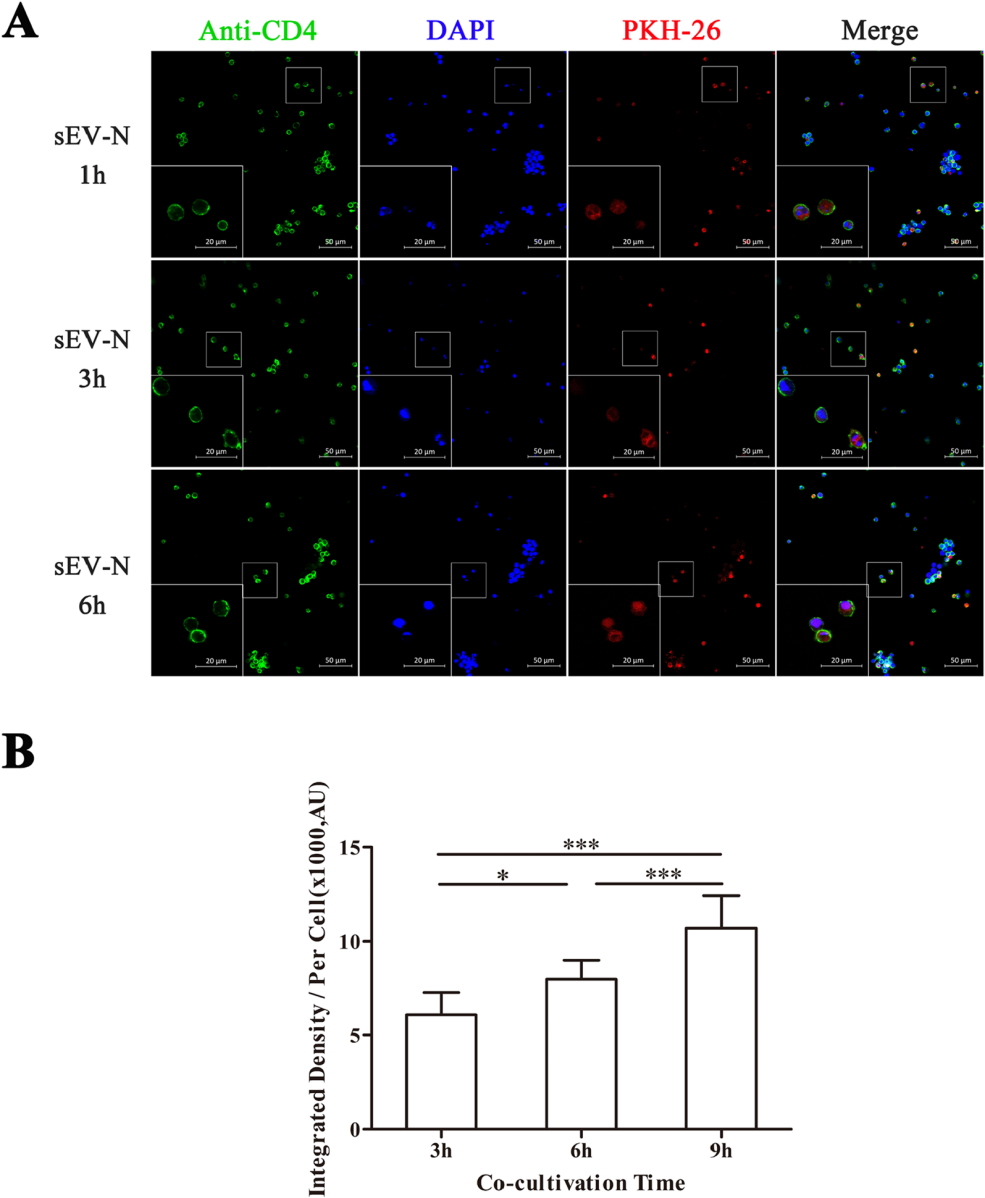
The distribution of sEVs, when cocultured with CD4 + T-cells for 1, 3, and 6 h in vitro at concentrations of 20 μg/ml*.* **A** Representative confocal images of PKH-26 immunofluorescence on the CD4 + cell. **B** The fluorescence intensity was measured by integrated density using ImageJ software. The mean integrated density of per cell increased significantly with incubation time. Mean ± SD, *n* = 3 per group, one-way ANOVA test. ***P < 0.01, *P < 0.05

It has been shown that sEVs function through internalization or binding to cell surface receptors [30]. To study the interaction pattern between sEVs and T-cells, we cocultured T-cells with PKH-26-labeled sEV-N (20 μg/mL), and collected cells at 1, 3, and 6 h to detect red fluorescence. We found the presence of red PKH-26 signal in CD4 + T-cells at 1, 3, and 6 h, and the fluorescence intensity of the cells increased with incubation time (Fig. 5A, B).

### Overexpression of IL10 enhanced the suppressive effect of mesenchymal stem cell-small extracellular vesicles on both the nonspecific proliferation and antigen-specific response of T-cells

**Fig. 6:**
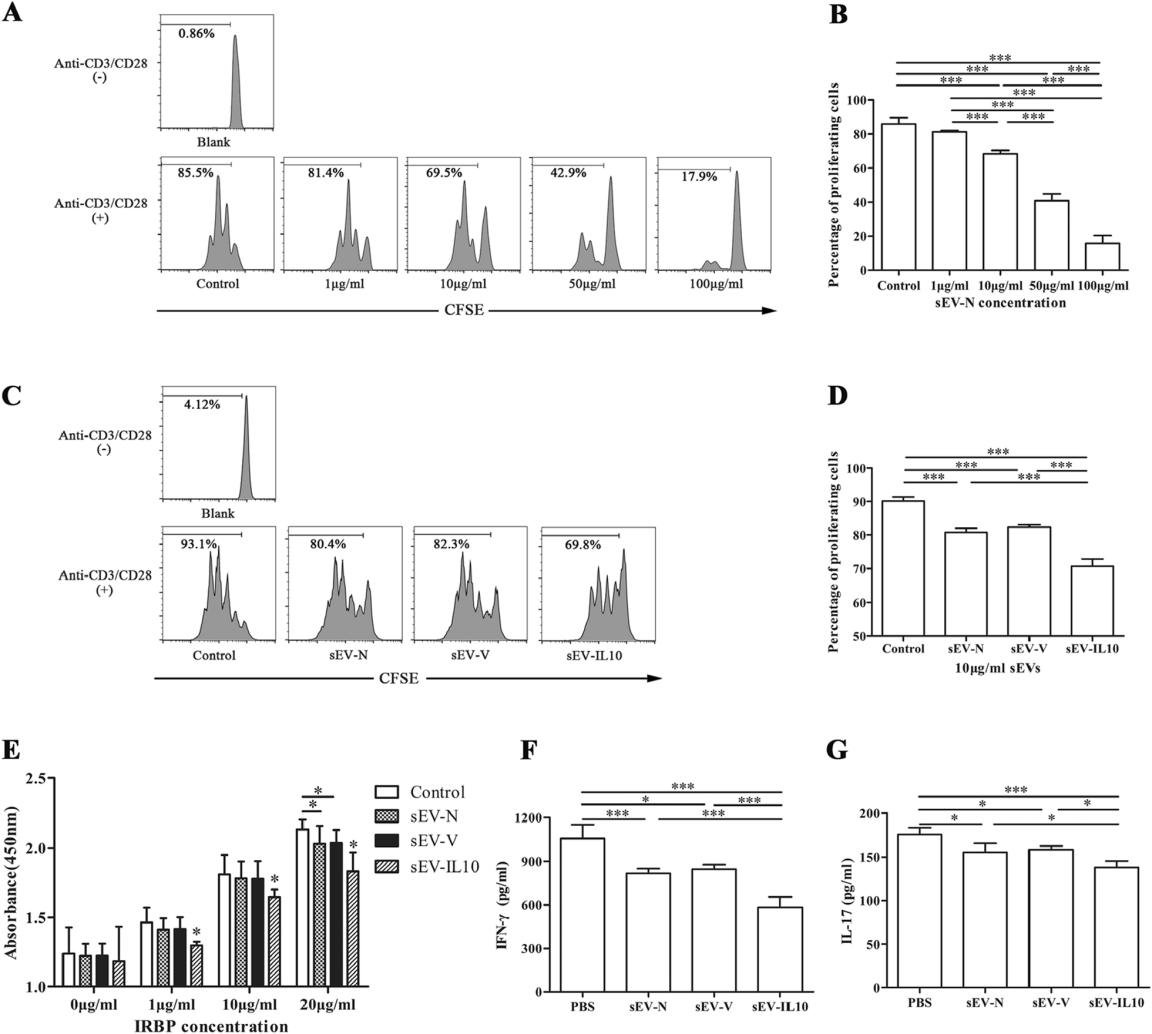
Inhibitory effects of sEVs derived from MSCs on the proliferation of T-cells. **A**, **B** sEV-N inhibit the proliferation of CD3/CD28-activated T-cells in a concentration-dependent manner. **C**, **D** sEV-IL10 enhance the suppressive effect of sEV-N on the proliferation of CD3/CD28-activated T-cells at concentrations of 10 μg/ml. **E.** Inhibitory effects of sEVs on antigen-specific T-cell responses. **F**, **G** Secreted cytokine levels of IFN-γ and IL17A in 20 μg/ml IRBP_651–670_-stimulated culture supernatants were determined by ELISA. Mean ± SD, one-way ANOVA test. ***P < 0.01, *P < 0.05

To identify the role of sEV-IL10 in the proliferation of T-cells, we verified the effects of different concentrations (0, 1, 10, 50, and 100 μg/mL) of sEV-N on the proliferation of naive CD4 + T-cells. Then, we labeled isolated T-cells from naïve mice with carboxyfluorescein succinimidyl ester (CFSE) and stimulated them with anti-CD3 and anti-CD28 antibodies. We found that sEV-N significantly inhibited the proliferation of CD4 + T-cells in a concentration-dependent manner (Fig. 6A, B).

Then, we compared the inhibitory capacity of the 3 sEV groups (10 μg/mL) on the proliferation of CD4 + T-cells. We found that sEV-IL10 exhibited the strongest inhibition among the 3 sEVs (Fig. 6C, D). We further assessed the inhibitory capacity of sEVs on the proliferation of IRBP_651–670_-specific T-cells using the BrdU kit. We isolated T-cells from the spleen and LNs of EAU mice (day 14 post-immunization) and then stimulated them with increasing doses of IRBP_651–670_. As shown in Fig. 6E, sEV-IL10 significantly inhibited the proliferation of IRBP651–670 specific T-cells (*P* < 0.05). Whereas, we found that the sEV-N and sEV-V groups showed significant inhibitory effects only at 20 μg/mL IRBP_651–670_ (*P* < 0.05). To compare IRBP_651–670_ specific Th1 and Th17 responses, the cell culture supernatants (with 20 μg/mL IRBP_651–670_) were collected and levels of IFN-γ and IL-17A were measured. As shown in Fig. 6G, F, the levels of IFN-γ and IL-17A were significantly lower in culture supernatants of sEV-IL10 treated T cells (*P* < 0.01). Our results indicated that IL-10-overexpressing sEVs greatly enhanced the suppressive effect of sEVs on both nonspecific and antigen-specific T-cell responses and Th1 and Th17 differentiation.

### Overexpression of IL10 enhanced the suppressive effect of mesenchymal stem cell-small extracellular vesicles on the differentiation of Th1 and Th17 cells and upregulated Treg cells in vitro

**Fig. 7:**
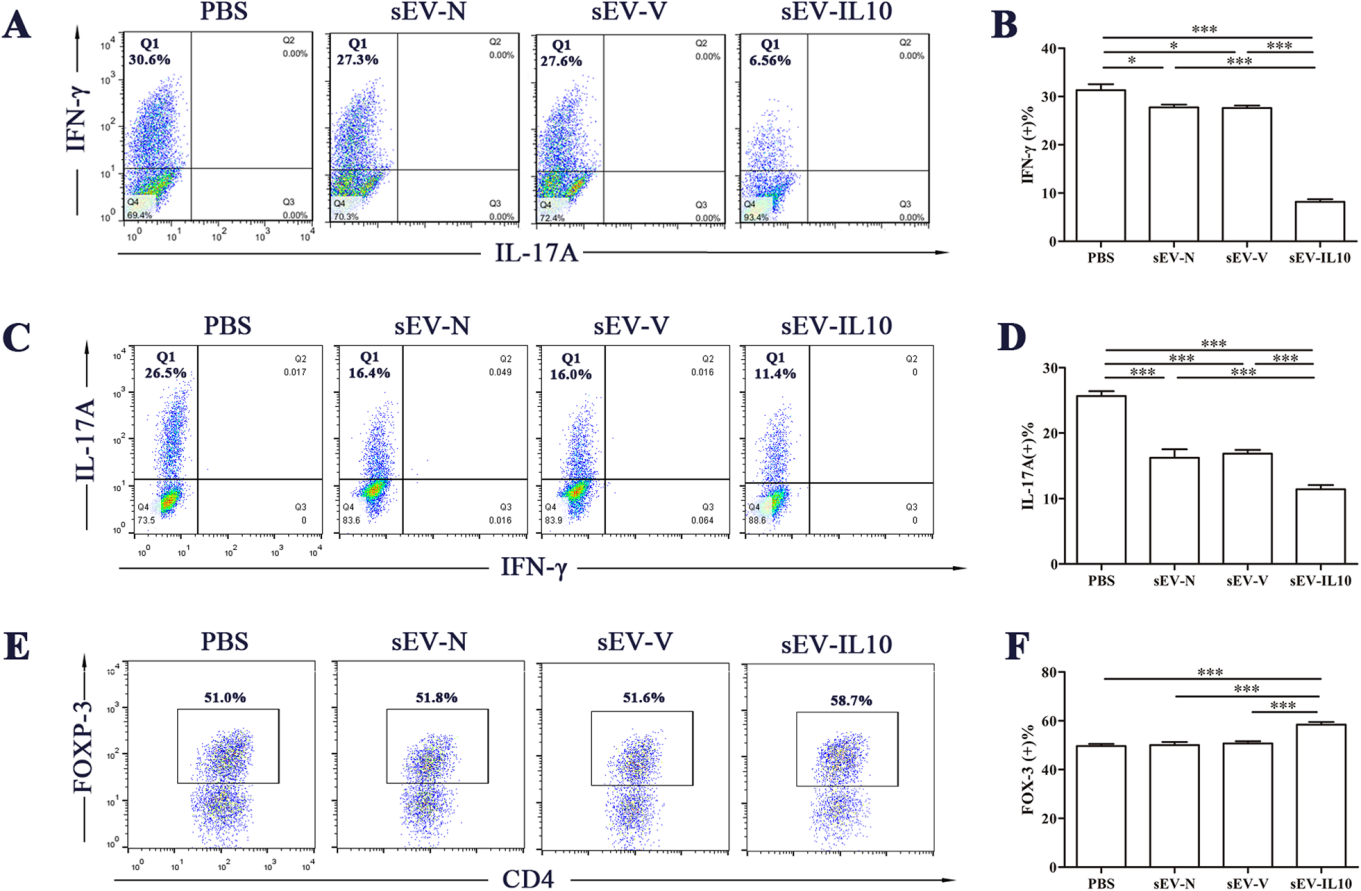
The effect of sEVs (10 μg/mL) on the differentiation of Th1, Th17, and Treg cells in vitro. **A**, **B** sEV-IL10 inhibit the differentiation of Th1 cells in vitro. **C**, **D** sEV-IL10 inhibit the differentiation of Th17 cells in vitro. **E**, **F** sEV-IL10 promote the differentiation of Th17 cells in vitro. Mean ± SD, one-way ANOVA test. ***P < 0.01, *P < 0.05

We further investigated the effect of sEV-IL10 on the T-cells differentiation. We isolated CD4 + T-cells from the spleen of naïve mice using a magnetic bead negative selection kit and incubated them with 10 μg/mL sEVs under Th1, Th17, and Treg cell differentiation conditions. After 5 d of culture, we evaluated cell differentiation using flow cytometry. We specifically observed that sEV-N and sEV-V inhibited the differentiation of both CD4 + IFN-γ + and CD4 + IL-17A + cells, while sEV-IL10 greatly enhanced the inhibitory effect of MSC-sEVs (*P* < 0.05) (Fig. 7A–D). We also found that under Treg cell differentiation conditions, sEV-IL10 upregulated the percentage of Fox3 + cells (*P* < 0.05), whereas sEV-N and sEV-V had no effect (Fig. 7E, F). These results indicated that the overexpression of IL-10 enhanced the immunosuppressive effect of sEVs on EAU by inhibiting the responses of both Th1 and Th17 cells and promoting the responses of Treg cells*.*

## Discussion

In this study, we established IL-10-overexpressing MSCs by transfecting them with a lentivirus carrying the *Il-10* gene and then obtained their sEVs, which contained high levels of IL-10 (sEV-IL10). These IL-10-overexpressing sEVs demonstrated long-lasting therapeutic effects on EAU compared with normal MSC-sEVs, which express low levels of IL-10. In vitro experiments revealed that overexpression of IL-10 greatly enhanced the suppressive effect of normal MSC-sEVs on the proliferation of T-cells and responses of Th1/Th17 cells, whereas upregulated Treg cells.

MSC-EVs have been reported to regulate the activation, proliferation, differentiation, maturation, and migration of various immune cells, exhibiting therapeutic potential in animal models of various immune-related diseases, such as inflammatory bowel disease (IBD), graft-versus-host disease (GVHD), collagen-induced arthritis (CIA), and autoimmune uveitis [24, 31–36]. MSC-EVs express several adhesion molecules, such as CD29, CD73, and CD44, which enable them to access the inflammatory and injury sites [37, 38], and transfer their contents to target cells to alter cell functions. We previously demonstrated that periocular injection of MSC-sEVs on established EAU attenuated disease progression in a rat model of EAU. However, the therapeutic effect was weak and multiple injections of MSC-sEVs were required [24]. Shigemoto-Kuroda et al. reported that preventive i.v. injection of 30 μg sEVs derived from human bone marrow MSCs at the same time of disease induction alleviated the progression of EAU [36]. However, in the clinical practice, patients usually visit to hospital after clinical symptoms occur and the therapeutic effect of sEVs on ongoing disease needs verified. Thus, using the current forms of MSC-sEVs to treat patients with uveitis or other autoimmune diseases is far from ideal. Therefore, improving the treatment efficacy of MSC-sEVs by reprogramming the encapsulated components of the vesicles is necessary for their potential clinical translation.

IL-10 is a well-known cytokine possessing a strong immunosuppressive effect; however, its use as an immunosuppressant for treating autoimmune diseases in the clinical settings has been limited by its short terminal half-life (approximately 2.7–4.5 h) [39] and rapid clearance in vivo. Due to their nanoscale size and long half-life, sEVs can be efficiently used as drug carriers [40–42]. The nanotoxicity and rapid drug clearance by living immune system limits the widespread application of nano-delivery systems and polyethyleneglycol (PEG) [43, 44]. In contrast, sEVs derived from the body have better biocompatibility and lower immunogenicity, and it was shown that their immune privileged status that can efficiently reduce drug clearance[45–47]. In addition, sEVs have the ability to cross biological barriers, such as the blood–brain and blood–ocular barriers [48–50]. Moreover, the lipid bilayer membrane of sEVs can protect the encysted proteins and miRNAs from degradation, thereby preserving their bioactivity for a long time [20, 51]. Our studies incorporating IL-10 into sEVs of MSCs enhanced the advantages of both IL-10 and sEVs of MSCs in the inhibition of immune suppression in autoimmune diseases. Compared with normal sEVs from MSCs, IL-10-overexpressing MSC-sEVs significantly inhibited EAU after a single i.v. injection at disease onset. These results indicated that MSC-sEVs carrying high concentrations of IL-10 possess strong and long-lasting immunosuppressive effects and are ideal cell-free candidates for treating autoimmune uveitis and potentially other autoimmune diseases.

As previously mentioned, due to their immunosuppressive effects, MSC-sEVs have been used for the inhibition of autoimmune responses. MSC-sEVs have been reported to reduce the differentiation of Th1 cells and production of IFN-γ by modulating the miRNA profile in Th1 cells in vitro and promoting the shift from Th1 to Th2 cells [52]. MSC-sEVs have also been shown to modulate the Th17/Treg balance by inhibiting the differentiation of Th17 cells and expression of IL-17 [53–55]. However, the role of MSC-sEVs in the proliferation of T-cells in vitro has been controversial. Studies by Chen and Andrade revealed that MSC-sEVs failed to suppress the proliferation of T-cells in vitro [56, 57], whereas others showed the opposite results [58–60]. Our in vitro results demonstrated that normal MSC-sEVs inhibited the proliferation of T-cells in a concentration-dependent manner and suppressed the differentiation of Th1 and Th17 cells. Moreover, we found that the IL-10-overexpressing MSC-sEVs exhibited superior advantages in inhibiting the proliferation of T-cells and differentiation of Th1 and Th17 cells, whereas upregulated the differentiation of Treg cells over normal sEVs. These results demonstrated a synergistic suppressive effect of both MSC-sEVs and IL-10 on the proliferation of T-cells and differentiation of Th1/Th17/Treg cells.

We also detected the presence of MSC-sEVs in the eyes and lymphoid tissues of EAU mice, in consistency with studies reporting that sEVs can cross the biological barrier [48] and MSC-sEVs possess chemotactic effects in diseased, but not naïve organs. For instance, Riazifar et al. detected the fluorescent signal of MSC-sEVs in the spinal cords of EAU but not WT mice [61]. Mao et al. found that only the colon tissues of IBD mice injected with indocyanine green labeled MSC-sEVs showed strong fluorescence [62]. It has been known that sEVs interact with target cells via various routes, such as internalization, binding to receptors on cell surface, and fusing with cell membrane [63, 64]. We found that sEVs were translocated in the cytoplasm of T-cells when cocultured in vitro, indicating that sEVs might exert their immunoregulatory effect directly on T-cells.

## Conclusions

In summary, our study demonstrated that sEVs derived from IL-10-overexpressing MSCs have notable effects on alleviating EAU development in mice. Thus, MSC-sEVs with high expression of IL-10 might be a novel approach for the treatment of autoimmune uveitis or other autoimmune diseases in human.

## Supplementary Information

**Additional file 1: Figure S1.** MSCs phenotype and differentiation capacity identification. **A** Positive expression of the CD29, and CD90 markers, and negative expression of the CD34 and CD45 markers. **B** Multilineage differentiation potential of MSCs.

**Additional file 2: Figure S2.** The bright field and GFP fluorescence photographs of MSC transduced with lentivirus.

## Data Availability

The datasets used or analyzed during the current study are available from the corresponding author on reasonable request.

## Abbreviations

sEVs: Small extracellular vesicles
MSCs: Mesenchymal stem cells
sEV-IL10: IL-10-overexpressing MSC-sEVs
EAU: Experimental autoimmune uveitis
LN: Lymph nodes
TNF-α: Anti-tumor necrosis factor-α
MSC-sEVs: MSC-derived sEVs
IL-10: Interleukin 10
ARVO: Association for research in vision and ophthalmology
TMUEC: Tianjin Medical University Eye Hospital
SPF: Specific pathogen-free
HEK-293 T: Human embryonic kidney 293 T cells
MOI: Multiplicity of infection
IFA: Incomplete Freund’s adjuvant
PTX: Pertussis toxin
sEV-N: Normal MSC-sEVs
sEV-V: Vector-infected MSC-sEVs
H&E: Hematoxylin and eosin
OCT: Optimal cutting temperature
i.v.: Intravenous injection
LNs: Lymph nodes
CFSE: Carboxyfluorescein succinimidyl ester
IBD: Inflammatory bowel disease
CIA: Collagen-induced arthritis
GVHD: Graft-versus-host disease
