# Supplementary figures and images for: Effect of small extracellular vesicles derived from IL-10-overexpressing mesenchymal stem cells on experimental autoimmune uveitis
Source: Stem Cell Res Ther. 2022 Mar 7;13:100. doi: 10.1186/s13287-022-02780-9 (PMC8900327; doi:10.1186/s13287-022-02780-9)

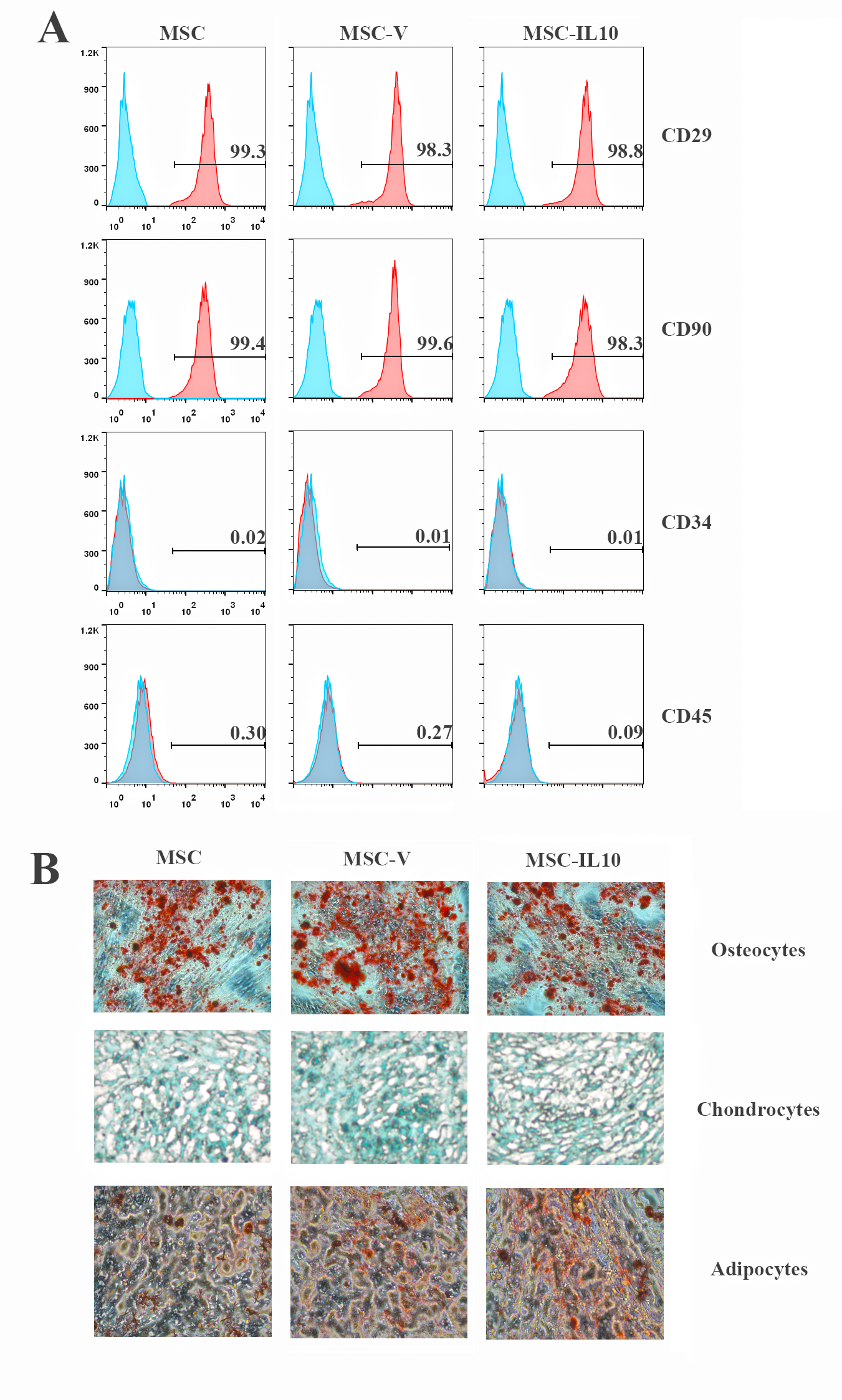

Supplement: Supplementary file 1 — Additional file 1: Figure S1. MSCs phenotype and differentiation capacity identification. A Positive expression of the CD29, and CD90 markers, and negative expression of the CD34 and CD45 markers. B Multilineage differentiation potential of MSCs. [file 13287_2022_2780_MOESM1_ESM.tif]

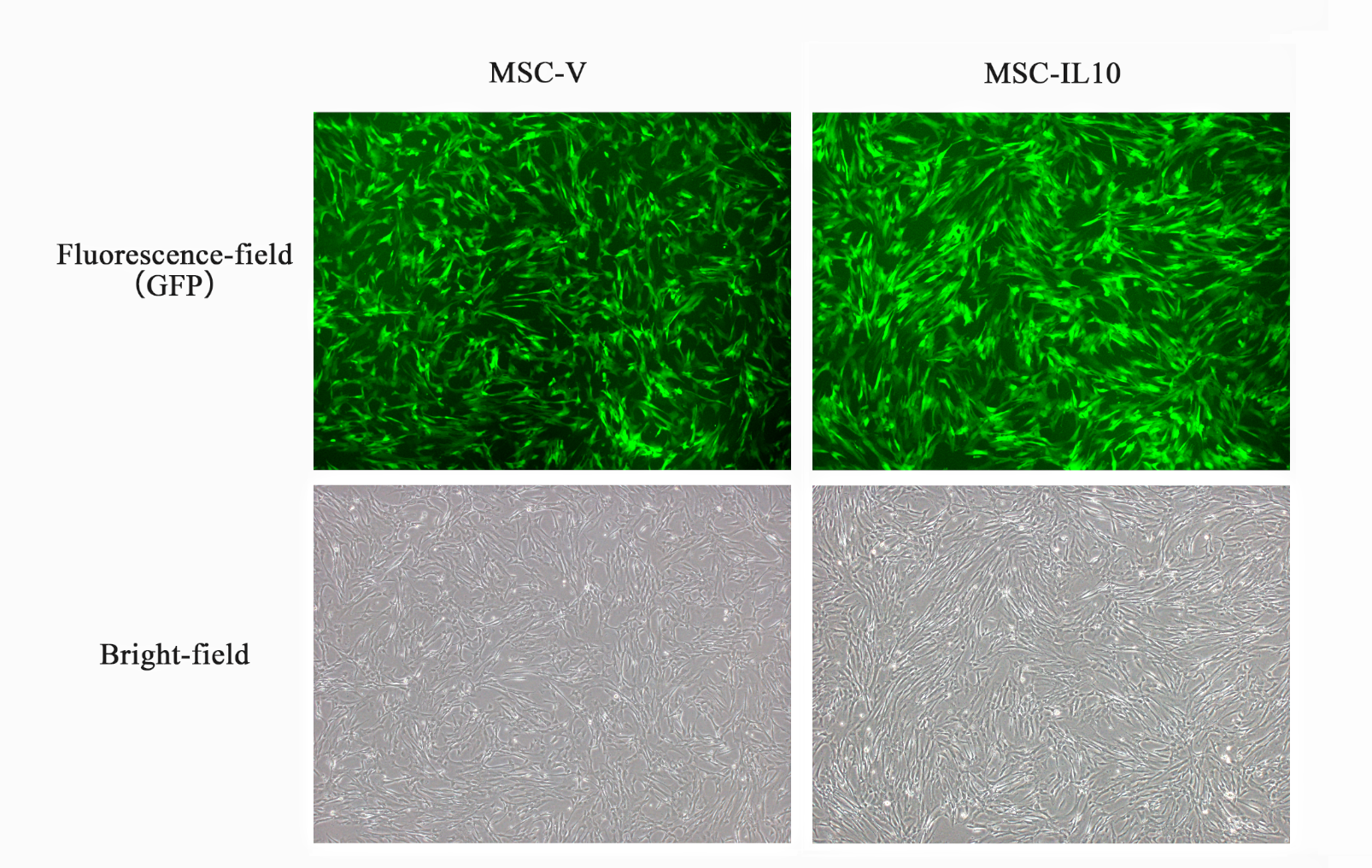

Supplement: Supplementary file 2 — Additional file 2: Figure S2. The bright field and GFP fluorescence photographs of MSC transduced with lentivirus. [file 13287_2022_2780_MOESM2_ESM.tif]
